# Animal welfare and farming systems synergistically influence beef cattle health: Evidence from Northern Thailand

**DOI:** 10.14202/vetworld.2025.3002-3016

**Published:** 2025-10-14

**Authors:** Nattamaporn Kongmuang, Payungsuk Intawicha, Choke Sorachakula, Somchart Tana, Wilasinee Inyawilert, Amornrat Wanangkarn, Sureeporn Saengwong

**Affiliations:** 1 Division of Animal Science, School of Agriculture and Natural Resources, University of Phayao, Phayao, 56000, Thailand; 2 Department of Agricultural Science, Faculty of Agriculture Natural Resources and Environment, Naresuan University, Phitsanulok, 65000, Thailand

**Keywords:** animal welfare, beef cattle, farming systems, health outcomes, PERMANOVA, Thailand

## Abstract

**Background and Aim:**

Beef cattle production in Thailand is vital for food security and rural livelihoods, yet differences in farming systems raise concerns about animal welfare and health. This study aimed to evaluate the interactive effects of animal welfare and farming systems on cattle health outcomes, providing insights for sustainable smallholder production.

**Materials and Methods:**

A cross-sectional study was conducted on 60 farms in Phayao Province, categorized as extensive, semi-intensive, or intensive. Animal welfare was assessed using an adapted Welfare Quality protocol with 41 indicators across five domains. Health outcomes were obtained from farm records and direct observations. Statistical analyses included Kruskal–Wallis tests, Dunn’s *post hoc* tests, Spearman’s rank correlation, and permutational multivariate analysis of variance with principal coordinate analysis.

**Results:**

Welfare scores differed significantly across systems, with intensive farms achieving the highest scores and extensive farms the lowest (p < 0.001). High-welfare farms showed reduced mortality, diarrhea, respiratory distress, bloating, parasitic infestation, and injuries compared with low-welfare farms (p < 0.05). Body condition score was strongly associated with welfare level (ρ = 0.68, p < 0.001). Multivariate analysis revealed significant effects of welfare level (R^2^ = 0.1787, p < 0.001), farming system (R^2^ = 0.1382, p = 0.0003), and their interaction (R^2^ = 0.2151, p = 0.0004) on cattle health. Semi-intensive farms with moderate welfare levels showed the most consistent and stable health outcomes.

**Conclusion:**

Animal welfare and farming systems interact to shape cattle health outcomes in Northern Thailand. Moderate welfare levels in semi-intensive systems offered balanced and consistent improvements, highlighting a scalable model for smallholders. The findings emphasize the need for context-specific welfare interventions, policy integration, and capacity-building initiatives to enhance both cattle health and farm sustainability.

## INTRODUCTION

Beef cattle production is a cornerstone of Thailand’s agricultural economy, contributing substantially to national food security and the livelihoods of rural communities. In 2024, the sector comprised 1,434,535 farmers managing approximately 9.9 million cattle, reflecting an annual increase of 248,657 head (2.58%) compared with the previous year [1]. Domestic demand remains exceptionally high, with consumption reaching 1.26 million head annually, while national production lags slightly at 1.19 million head [2]. The Northeastern and northern regions serve as the country’s main production areas due to favorable conditions for free-grazing and traditional husbandry practices. Farmers in these regions predominantly employ extensive systems that rely on natural pastures to minimize feed costs [3]. Although extensive farming offers cost advantages, it is often associated with inadequate nutritional and health management, resulting in slower growth, nutrient deficiencies, and heightened vulnerability to infectious diseases in uncontrolled environments. Limited knowledge of biosecurity, disease control, and food safety standards among many farmers further constrains the development of efficient production practices [4]. While semi-intensive and intensive systems have been introduced, structural and resource-related barriers continue to restrict their widespread adoption.

Beyond production challenges, ethical concerns surrounding intensive livestock management have intensified public scrutiny of cattle production practices, particularly in relation to animal welfare and sustainability. Intensive methods are frequently criticized for compromising animals’ physical and psychological well-being, leading to stress, discomfort, and restricted expression of natural behaviors that are essential for overall welfare [5]. Animal welfare, defined as the physical and mental well-being of animals achieved through adequate nutrition, disease prevention, behavioral expression, and humane handling, directly influences cattle health and productivity [6]. It affects key outcomes such as meat quality, disease resistance, and reproductive performance, and plays a pivotal role in enhancing the sustainability and profitability of production systems [7]. With rising consumer awareness, animal welfare has emerged as a central concern among producers, consumers, and policymakers, shaping perceptions of product quality, farm credibility, and the broader sustainability of agricultural systems [8].

Despite the economic importance of beef cattle production in Thailand, particularly in the northern and northeastern regions, empirical evidence linking animal welfare to health outcomes within different farming systems remains limited. Previous studies have primarily focused on describing production systems or evaluating welfare and health independently, without accounting for their interrelationships. Most investigations have relied on univariate approaches that fail to capture the complex interactions between welfare domains, farming systems, and animal health outcomes. Furthermore, available welfare assessment protocols such as the Welfare Quality^®^ assessment protocol for cattle (Welfare Quality^®^ Consortium, Lelystad, The Netherlands, 2009) have seldom been adapted and validated for smallholder conditions in tropical contexts, where structural limitations, resource constraints, and traditional practices prevail [9]. This knowledge gap is particularly critical in Thailand, where extensive systems remain dominant but are associated with inadequate health and nutritional management. While semi-intensive and intensive systems are being promoted as alternatives, their adoption has been slow, and their welfare and health implications are not well documented. Moreover, limited attention has been given to consumer-driven concerns regarding animal welfare and sustainability, despite these issues increasingly shaping the credibility and marketability of livestock products. Thus, there is a need for integrated, evidence-based analyses that examine how farming systems and welfare conditions jointly influence cattle health in smallholder production settings.

This study was designed to address these gaps by systematically examining the interactive effects of farming systems and animal welfare on the health outcomes of beef cattle in Northern Thailand. Specifically, it aimed to (i) compare animal welfare scores across extensive, semi-intensive, and intensive systems; (ii) evaluate differences in cattle health outcomes across farms with low, moderate, and high welfare levels; (iii) identify associations between welfare scores and individual health indicators; and (iv) determine the combined effects of farming systems and welfare levels on multivariate health outcomes using advanced statistical approaches. By integrating welfare assessment protocols with health outcome monitoring, the study provides empirical evidence tailored to smallholder contexts. The findings are expected to inform welfare-oriented policies, guide farmer training and capacity building, and support the development of scalable, cost-effective strategies that enhance both animal health and production sustainability in Thailand and comparable tropical systems.

## MATERIALS AND METHODS

### Ethical approval and Informed consent

The study protocol received approval from both the Human Ethics Committee (UP-HEC 1.2/030/66) and the Animal Ethics Committee (UP-AE: 1-034-65) of the University of Phayao. Following these approvals, a household survey was conducted, involving in-person interviews with 60 beef cattle farmers. All participants were informed about the study’s objectives, data collection procedures, and confidentiality measures, and provided written informed consent before participation.

### Study period and location

**Figure 1:**
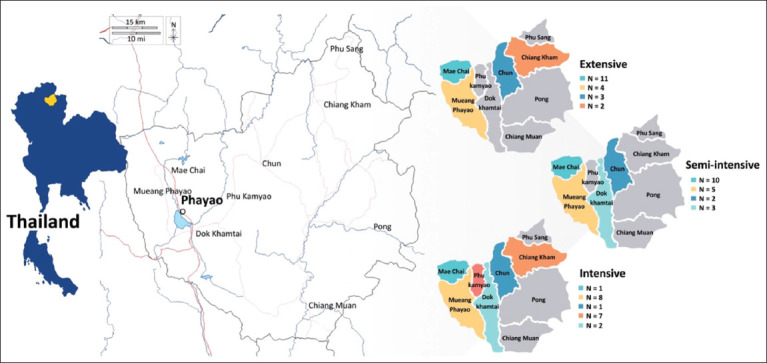
Map of the study area and the spatial distribution of beef cattle farms in Phayao Province. The pictures on the left show the location of Phayao Province within Northern Thailand. The right pictures present district-level farm counts for extensive (n = 20), semi-intensive (n = 20), and intensive (n = 20) systems, with each district color-coded and labeled. The numbers indicate the total number of farms surveyed in each district [Source: https://www.d-maps.com/carte.php?num_car=299253&lang=en].

This cross-sectional study was conducted between October 2023 and September 2024 in Phayao Province, Northern Thailand (19.1710° N, 99.9067° E), which spans 6,335 km^2^ with elevations ranging from 300 to 1,500 m above sea level. The region experiences a tropical climate with three distinct seasons: Rainy (May–October), cool dry (November–February), and hot dry (March–April). Average temperatures range from 17°C in January to 35°C in April, with annual rainfall of 1,100–1,400 mm. Farm locations were recorded at the district level and mapped by production system type (extensive, semi-intensive, intensive; 20 farms each) to show their spatial distribution (Figure 1).

### Conceptual framework

**Figure 2:**
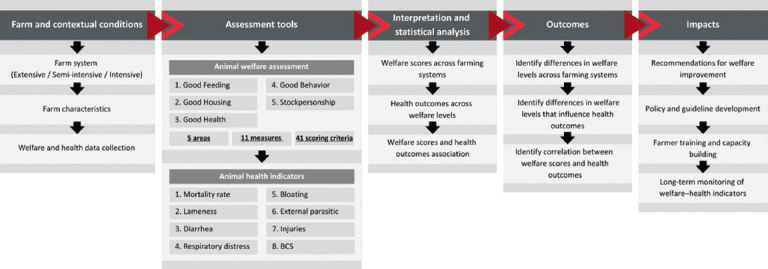
Conceptual framework of the study: pathways linking farming systems, animal welfare assessment, health outcomes, and policy implications.

The conceptual framework (Figure 2) illustrates the logical pathways linking farm conditions, animal welfare, and health outcomes across smallholder beef production systems. The farming system type (extensive, semi-intensive, or intensive) and farm characteristics served as the contextual basis, generating two primary datasets: welfare scores derived from adapted indicators, and health outcomes assessed through farm records and observations. Comparative and correlational analyses were then conducted to evaluate differences in welfare and health outcomes across systems. This framework highlights measurable links between welfare domains and cattle health status, providing an evidence-based foundation to support policy recommendations, farmer training, welfare guidelines, and long-term monitoring in smallholder production contexts.

### Farm selection and classification

Sixty farms were selected to ensure equal representation across production systems. Eligibility criteria required at least five cattle and a minimum of 2 years’ farming experience. Farming systems were defined as follows:

- Extensive: cattle kept outdoors on pasture year-round with occasional shelter
- Semi-intensive: cattle provided 6–10 h of daily grazing supplemented with additional feed
- Intensive: cattle confined to pens without pasture access, fully dependent on human provision of food, water, and shelter.

A sensitivity analysis using G*Power (α = 0.05; 1–β = 0.80) confirmed that a sample size of 60 farms was sufficient to detect medium–large differences (f ≈ 0.41) in welfare scores and health outcomes across systems.

### Animal welfare assessment

Animal welfare was assessed using a structured protocol adapted from Welfare Quality [9], Gottardo *et al*. [10], and Kaurivi *et al*. [11], modified for local Thai conditions. The protocol emphasized ease of use, avoidance of animal harm, clarity of measures, and applicability in field settings without laboratory testing. Indicators impractical in smallholder contexts, such as ventilation, ammonia concentration, or quarantine pens, were excluded. Validity was established through expert review by three specialists, yielding a content validity index of 0.91.

The protocol encompassed five domains (Good Feeding, Good Housing, Good Health, Good Behavior, Good Stockpersonship), covering 11 measures and 41 indicators (Supplementary Table S1). Farms were classified into welfare levels based on total scores:

- High welfare: 100–116 points (≥86.2%)
- Moderate welfare: 75–99 points (64.7%–85.3%)
- Low welfare: 41–74 points (35.3%–63.7%)

### Health outcome evaluation

Health data were obtained from farm records and direct observations, including annual mortality, lameness, respiratory distress, diarrhea, bloating, external parasitism, injuries, and body condition score (BCS, 1–5 scale).

### Data collection

Two trained observers collected welfare and health data using standardized scoring manuals and anchor examples. Inter-observer consistency was verified through discussion and agreement prior to statistical analysis. Farm records were cross-verified with observations to ensure accuracy. Data collection spanned 12 months, covering all three climatic seasons to minimize seasonal bias.

### Statistical analysis

Data normality was assessed using the Shapiro–Wilk test. As most variables were non-normally distributed (p < 0.05), non-parametric methods were employed. Descriptive statistics (medians, interquartile ranges [IQRs]) summarized welfare and health outcomes.

- Kruskal–Wallis tests compared welfare scores across farming systems and health outcomes across welfare levels, with Dunn’s *post hoc* test (Bonferroni correction) for pairwise differences
- Spearman’s rank correlation assessed associations between welfare scores and individual health outcomes
- Permutational multivariate analysis of variance (PERMANOVA) (9,999 permutations, Bray–Curtis dissimilarity) tested main and interaction effects of welfare level and farming system on multivariate health outcomes. Homogeneity of dispersion was confirmed (p > 0.05)
- Principal coordinates analysis (PCoA) visualized dissimilarities among systems and welfare levels.

All analyses were conducted using the Statistical Package for the Social Sciences version 23.0 (IBM, Armonk, NY, USA) and R v4.4.3, with significance set at p < 0.05.

## RESULTS AND DISCUSSION

### Comparison of animal welfare scores across beef cattle farming systems

#### Distribution of welfare levels and scores

Table 1 presents the distribution of farms by welfare level and the descriptive statistics of total welfare scores across the three farming systems. The assessment indicated that 21 farms had poor welfare levels, 27 received a moderate score, and 12 achieved a high score. Most farms operating under the extensive system were classified as having low welfare levels, whereas semi-intensive farms tended to fall into the moderate category, reflecting higher welfare scores. Most farms in intensive farming systems demonstrated a high welfare standard, with some classified at a moderate level. Supplementary Table S2 provides detailed farm-level data for all 60 farms, including farming system, total welfare score, and welfare classification.

**Table 1:** Distribution of farms by welfare level (low, moderate, and high) and descriptive statistics (mean±SD, minimum–maximum) of total welfare scores across the three farming systems (extensive, semi-intensive, and intensive).

Farming system	Low n (%)	Moderate n (%)	High n (%)	Mean total welfare score ± SD	Welfare score (min–max)
Extensive	17 (80.95)	3 (11.11)	0 (0.00)	67.40±5.86	56.27–77.80
Semi-intensive	4 (19.05)	16 (59.26)	0 (0.00)	84.64±6.82	71.20–93.50
Intensive	0 (0.00)	8 (29.63)	12 (100.00)	100.28±4.66	90.50–108.38

SD = Standard deviation

Numerous studies have reported significant welfare issues relating to rearing systems, mainly because of poor nutritional strategies, poor parasite control, and exposure to severe or unpredictable weather conditions. These empirical data are consistent with the findings of Kaurivi *et al*. [12], who observed that the pen-fed cattle farm in Namibia was more compliant with the animal welfare standards than the other system, although it was measured by the standard criteria. Similarly, Temple and Manteca [13] and Williams *et al*. [14] identified critical inadequacies in husbandry practices, including variable feed quality, a lack of water supply, and poor health monitoring, as barriers to achieving welfare standards.

#### Statistical comparison across systems

Table 2 presents the Kruskal–Wallis test results, which compare welfare scores across the three farming systems. The analysis revealed statistically significant differences in all five welfare aspects, namely Good Feeding, Good Housing, Good Health, Good Behavior, and Stockpersonship, as well as the total welfare score (χ^2^ = 50.30, p < 0.001, ε² = 0.85). The median scores and IQRs indicate that the intensive system had the highest overall welfare score, followed by the semi-intensive system, whereas the extensive system had the lowest score in almost all welfare factors. The findings indicate a strong trend in welfare performance in accordance with the degree of farming intensification.

**Table 2:** Welfare scores across the three farming systems.

Welfare aspects	Extensive (n = 20)		Semi-intensive (n = 20)		Intensive (n = 20)		Chi-square	p-value	ε²
		
	Mean rank	Median (IQR)	Mean rank	Median (IQR)	Mean rank	Median (IQR)			
Good feeding	15.35	9.08 (2.49)	28.60	11.71 (1.76)	47.55	14.00 (2.85)	34.36	<0.001	0.57
Good housing	14.18	13.50 (3.00)	27.88	17.50 (3.00)	49.45	22.00 (2.75)	41.73	<0.001	0.70
Good health	26.40	26.00 (2.75)	26.15	26.00 (2.00)	38.95	28.00 (3.00)	7.29	0.026	0.11
Good behavior	10.50	14.00 (1.00)	33.03	24.00 (4.75)	47.98	28.00 (1.75)	47.41	<0.001	0.80
Stockpersonship	12.70	5.00 (0.00)	28.85	7.00 (1.00)	49.95	9.00 (1.00)	48.52	<0.001	0.82
Total welfare score	11.25	66.78 (6.79)	29.85	87.17 (6.57)	50.40	100.90 (7.30)	50.30	<0.001	0.85
Welfare level	Low		Moderate		High				

IQR = Interquartile range

This observation supports previous studies, which have shown that more organized and resource-intensive systems tend to comply with animal welfare standards. The most common qualities of intensive systems are enhanced infrastructure, controlled feeding practices, and routine health assessments, all of which contribute to improved welfare outcomes [12, 13]. In contrast, extensive systems are likely to have an inconsistent supply of resources and inadequate stockperson supervision, which can be harmful to animal health and reduce the chances of natural behavioral expression. This highlights the importance of considering farming practices in relation to specific welfare requirements, particularly in systems with limited structural support.

#### Pairwise post hoc comparisons

Following the significant results of the Kruskal–Wallis test, Dunn’s *post hoc* test with Bonferroni correction was conducted to examine pairwise differences in welfare across the three farming systems. Table 3 shows that most welfare aspects, particularly Good Feeding, Housing, Behavior, Stockpersonship, and the Total Welfare Score, showed statistically significant differences in several pairwise comparisons among the systems (p < 0.05). For example, Good Feeding differed significantly between the extensive and semi-intensive systems (p = 0.049, |r| = 0.31), extensive and intensive systems (p < 0.001, |r| = 0.75), and semi-intensive and intensive systems (p = 0.002, |r| = 0.44). Similarly, Stockpersonship showed significant differences in all pairwise comparisons (p < 0.01). In contrast, Good Health showed no significant differences across any of the systems (p > 0.05), suggesting that factors such as individual animal care or disease prevalence may influence health outcomes more than farming system type alone. The total welfare score showed significant differences across all comparisons: extensive versus semi-intensive (p = 0.002, |r| = 0.43), extensive versus intensive (p < 0.001, |r| = 0.91), and semi-intensive versus intensive (p = 0.001, |r| = 0.48), indicating that the type of farming system has a strong influence on the overall welfare of beef cattle.

**Table 3:** Pairwise comparisons of welfare aspects across farming systems using Dunn’s *post hoc* test with Bonferroni correction (p = 0.05).

Welfare aspects	Ext versus semi-int	|r|	Ext versus Int	|r|	Semi-int versus Int	|r|
Good feeding	0.049*	0.31	0.000***	0.75	0.002**	0.44
Good housing	0.039*	0.32	0.000***	0.83	0.000***	0.51
Good health	1.000^ns^	0.01	0.062^ns^	0.30	0.055^ns^	0.30
Good behavior	0.000***	0.53	0.000***	0.88	0.019*	0.35
Stockpersonship	0.008**	0.39	0.000***	0.90	0.000***	0.51
Total welfare score	0.002**	0.43	0.000***	0.91	0.001***	0.48

*** p < 0.001,

** p < 0.01,

* p < 0.05,

ns p ≥ 0.05, Ext = Extensive, Int = Intensive

#### Interpretation of welfare comparisons

Pairwise comparisons highlighted the multifaceted nature of animal welfare, with each aspect responding differently to different farming systems. This variation underscores the importance of environmental and managerial factors, which is consistent with the findings of Linstädt *et al*. [15]. Most intensive systems are likely to score higher in welfare because of the control of environments, feeding, monitoring, and treatment procedures that may be routinely employed, as previously reported by Mota-Rojas *et al*. [16] and Hubbard *et al*. [17]. Although extensive systems provide animals with more access to the natural environment, lower evaluation results in feeding and housing suggest that limited resources and less human contact may negatively impact welfare. The significant differences observed in Good Behavior indicate that behavioral well-being is shaped not only by space and natural exposure but also by stockperson procedures and the quality of daily handling. Interestingly, no significant differences were observed in health outcomes across farm systems, suggesting that health-related indicators may be more evenly distributed among farms or influenced by external factors such as veterinary practices or regional disease pressure. The overall similarity of the total welfare scores in each comparison indicates a significant effect of the type of farming system on the overall welfare of the animals. However, the variability within individual welfare aspects suggests that system classification alone is insufficient to ensure improved welfare standards. Kannan and Lama [18] noted that the quality of farm management and investment in welfare-enhancing resources ultimately shape the lived experiences of animals, regardless of the production system.

### Evaluation of animal health outcomes across different welfare levels

#### Kruskal–Wallis results

Table 4 presents the results of the Kruskal–Wallis test, which assesses differences in health outcomes across low, moderate, and high welfare levels. Significant differences were observed in seven of the eight health outcomes: mortality rate (χ^2^ = 6.371, p = 0.041, ε² = 0.08), diarrhea (χ^2^ = 7.128, p = 0.028, ε² = 0.09), respiratory distress (χ^2^ = 6.973, p = 0.031, ε² = 0.09), bloating (χ^2^ = 17.208, p < 0.001, ε² = 0.27), external parasitic infestation (χ^2^ = 11.094, p = 0.004, ε² = 0.16), injuries (χ^2^ = 20.358, p < 0.001, ε² = 0.32), and BCS (χ^2^ = 28.363, p < 0.001, ε² = 0.46). Lameness was the only variable that did not show a statistically significant difference (χ^2^ = 0.779, p = 0.677, ε² = 0.00). These results indicate that higher welfare levels are generally associated with better health outcomes, particularly in reducing the incidence of disease, injury, and poor body condition.

**Table 4:** Comparison of health outcomes across the three welfare levels.

Health outcomes	Low (n = 21)		Moderate (n = 27)		High (n = 12)		Chi-square	p-value	ε²
		
	Mean rank	Median (IQR)	Mean rank	Median (IQR)	Mean rank	Median (IQR)			
Mortality rate	34.88	0.00 (2.00)	30.65	0.00 (1.00)	22.50	0.00 (0.00)	6.371	0.041	0.08
Lameness	32.14	1.00 (2.00)	30.80	1.00 (2.00)	26.96	0.00 (1.75)	0.779	0.677	0.00
Diarrhea	36.76	2.00 (3.50)	30.09	1.00 (3.00)	20.46	1.00 (2.00)	7.128	0.028	0.09
Respiratory distress	37.31	2.00 (3.00)	28.87	0.00 (2.00)	22.25	0.00 (0.75)	6.973	0.031	0.09
Bloating	41.90	4.00 (3.50)	27.41	2.00 (2.00)	17.50	1.00 (1.75)	17.208	0.000	0.27
External parasitic	37.90	5.00 (6.50)	30.72	3.00 (3.00)	17.04	1.00 (3.00)	11.094	0.004	0.16
Injuries	40.95	3.00 (9.50)	30.09	2.00 (3.00)	13.13	0.00 (0.00)	20.358	0.000	0.32
BCS	16.55	2.31 (0.43)	32.83	2.78 (0.60)	49.67	3.33 (1.02)	28.363	0.000	0.46

IQR = Interquartile range, BCS = Body condition score

#### Interpretation of health–welfare links

These findings are consistent with those of Cooke *et al*. [19], who emphasized that increased animal welfare is strongly linked to improved health, and this association can be attributed to enhanced management practices, improved environmental control, and positive human–animal interactions. Among the health variables, injuries and bloating showed particularly high χ^2^ values, indicating that these conditions are particularly responsive to changes in welfare practices. Winton *et al*. [20] stated that injuries and gastrointestinal problems often reflect the quality of stockpersonship, housing design, and stress mitigation strategies used on farms. Mechanistically, injuries often result from inadequate flooring, housing design, or rough handling, while bloating is closely linked to feeding practices and ruminal function. Both conditions are intensified under stress, which compromises immune and digestive physiology, thereby explaining their strong association with poor welfare [21, 22]. In addition, the significantly higher BCS observed in high-welfare farms supports the findings of Praveen *et al*. [23], who identified BCS as a reliable indicator of nutritional adequacy and overall herd management. The absence of significant differences in the prevalence of lameness across welfare categories is consistent with the findings of Morrone *et al*. [24] and Matshetsheni and Jaja [25], who suggested that mobility issues are more strongly influenced by factors such as ground surface, genetics, and flooring conditions than by welfare level alone. These findings underscore the importance of quality management at all levels of animal welfare, as suggested by Kannan and Lama [18]. Good management can help bridge structural gaps within farming systems and enhance animal health. Regardless of intensive systems, welfare-oriented practices are essential for preventing health issues and improving overall herd well-being.

Following the significant results from the Kruskal–Wallis test, Dunn’s *post hoc* test with Bonferroni correction identified significant pairwise differences in several health outcomes across welfare levels (Table 5). Notably, significant differences were observed between the low and high welfare groups for mortality rate (p = 0.035, |r| = 0.32), diarrhea (p = 0.023, |r| = 0.34), respiratory distress (p = 0.033, |r| = 0.33), bloating (p < 0.001, |r| = 0.44), external parasitic infestation (p = 0.003, |r| = 0.43), and injuries (p < 0.001, |r| = 0.59). These findings indicate a strong association between higher welfare standards and a reduced incidence of health problems, particularly those related to infection and injury. In addition, BCS showed clear differences across all three comparisons: between low and moderate levels (p = 0.004, |r| = 0.41), moderate and high levels (p = 0.016, |r| = 0.36), and low and high levels (p < 0.001, |r| = 0.68), underscoring its value as a sensitive indicator of nutrition and overall well-being. A higher BCS in well-managed farms reflects consistent feeding practices and adequate energy balance, which in turn support improved animal performance and resilience. Collectively, these results point to the essentiality of high welfare standards in promoting better health outcomes and optimal nutritional status in beef cattle.

**Table 5:** Pairwise comparisons of health outcomes across animal welfare levels using Dunn’s *post hoc* test with Bonferroni correction.

Health outcomes	Low versus moderate	|r|	Moderate versus high	|r|	Low versus high	|r|
Mortality rate	0.850^ns^	0.14	0.250^ns^	0.22	0.035*	0.32
Diarrhea	0.526^ns^	0.17	0.302^ns^	0.21	0.023*	0.34
Respiratory distress	0.228^ns^	0.23	0.729^ns^	0.15	0.033*	0.33
Bloating	0.011*	0.38	0.283^ns^	0.22	0.000***	0.44
External parasitic	0.462^ns^	0.18	0.068^ns^	0.29	0.003**	0.43
Injuries	0.086^ns^	0.28	0.012*	0.35	0.000***	0.59
BCS	0.004**	0.41	0.016*	0.36	0.000***	0.68

*** p < 0.001,

** p < 0.01,

* p < 0.05,

ns p ≥ 0.05. BCS = Body condition score

The pairwise comparison also indicated that there was a significant difference in injuries and bloating between the low and the high welfare groups. The findings are consistent with studies by De León *et al*. [26] and Nyangiwe and Matthee [27], which emphasized that regular hygiene practices and health monitoring are important to control the parasitic infection and physical injury. Similarly, the low prevalence of external parasites recorded in high-welfare farms highlights the importance of preventive health-care programs in enhancing and ensuring animal health. In summary, these findings confirm the fact that animal welfare has a positive effect on animal health. In addition to the ethical reasons, the positive returns on investing in animal welfare can be realized in the form of decreased disease incidents, decreased treatment expenses, and increased productivity, which should highlight the strategic value of welfare-focused farm management**.**

### Examination of the association between animal welfare scores and individual health outcomes

#### Correlation coefficients

**Figure 3:**
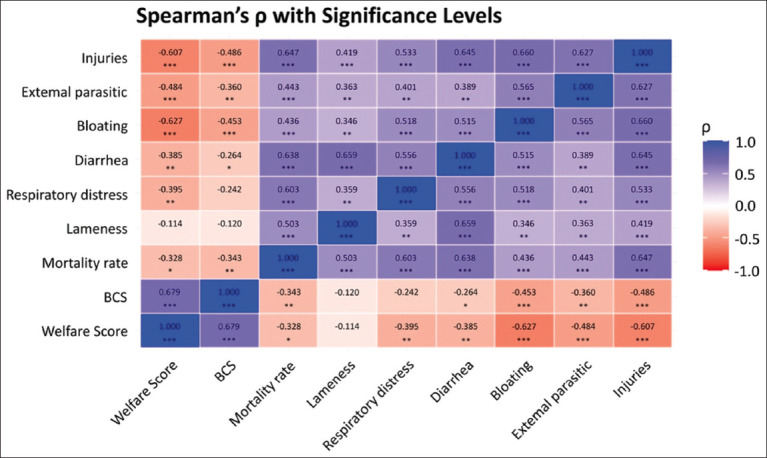
Heatmap of Spearman’s rank correlation coefficients (ρ) and corresponding p-values between animal welfare scores and individual health outcomes. The strength and direction of correlations are color-coded, with statistically significant associations (p < 0.05) highlighted to support the interpretation of relationships among variables.

Figure 3 presents the Spearman’s rank correlation coefficients (ρ) between welfare scores and individual health outcomes. The BCS showed a moderate positive correlation with the total welfare score (ρ = 0.68, p < 0.001), highlighting its central role in distinguishing the welfare status of animals across farms. In addition, BCS was moderately negatively correlated with bloating (ρ = −0.45, p < 0.001) and injuries (ρ = −0.49, p < 0.001), emphasizing its value as a comprehensive indicator of physical health and nutritional adequacy. These findings support previous research that identified BCS as a key welfare indicator reflecting nutrition, health, and physiological resilience under various farm management conditions [28–31].

#### Negative and positive associations

Total welfare scores were moderately negatively correlated with bloating (ρ = −0.63, p < 0.001) and injuries (ρ = −0.61, p < 0.001), suggesting that these variables may serve as reliable indicators of lower welfare status. Several health outcomes were moderately positively correlated, including mortality and injury (ρ = 0.65, p < 0.001), lameness and diarrhea (ρ = 0.66, p < 0.001), respiratory distress and mortality (ρ = 0.60, p < 0.001), bloating and injury (ρ = 0.66, p < 0.001), and infestation and injury caused by external parasites (ρ = 0.63, p < 0.001). These correlations indicate that such conditions often co-occur, reflecting systemic issues rather than isolated incidents.

#### Broader management challenges

The co-occurrence of health problems underscores broader management and environmental challenges, highlighting the need for an integrated approach to assess welfare indicators [15, 22]. The common features of these systems include dependence on low-input practice, seasonal feed limitation, and parasitic and heat stress susceptibility. The association between the welfare condition and health in cattle observed in our analysis is likely to have wider application in these smallholder systems within the tropics. Therefore, the experiences in this research are the basis behind the establishment of evidence-based, low-cost decision-supporting tools to augment welfare monitoring and increase herd productivity in smallholder settings [28, 32].

### Evaluation of the main and interaction effects of animal welfare level and farming system on multivariate animal health outcomes

#### PCoA based on welfare levels

**Figure 4:**
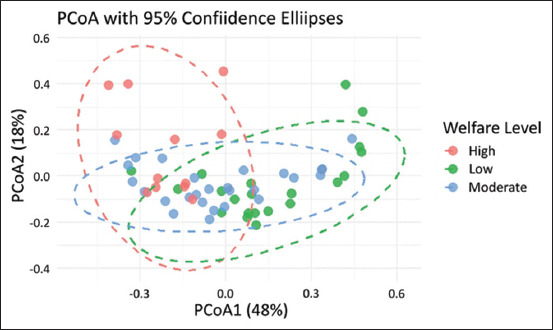
Principal component analysis plot based on Bray–Curtis dissimilarity showing health outcome distributions across welfare levels with 95% confidence ellipses, illustrating partial separation of high-welfare farms with more consistent clustering, while low- and moderate-welfare farms show considerable overlap.

Figure 4 illustrates that the PCoA was based on Bray–Curtis dissimilarity, showing the multivariate distribution of animal health outcomes across different animal welfare levels. The first axis (PCoA1) explained 48% of the total variation, whereas the second axis (PCoA2) explained 18%, providing a meaningful visual assessment of group differences. The tight clustering observed in the high-welfare group (red) indicates lower within-group variability, likely reflecting the farms’ ability to maintain consistent management practices and stable environmental conditions, such as improved nutrition, environmental control, effective health care, and skilled stockmanship, which support stable and favorable animal health and welfare outcomes, even under challenging conditions [33]. In contrast, the broader dispersion observed in the low (green) and moderate (blue) welfare groups, particularly along the PCoA1 axis, may indicate inconsistent management, transitional welfare states, and farm-specific health issues. These findings align with those of Main *et al*. [34], who noted that farms rarely performed uniformly well or poorly across all welfare indicators, with each farm exhibiting its own pattern of challenges.

#### PCoA based on farming systems

**Figure 5:**
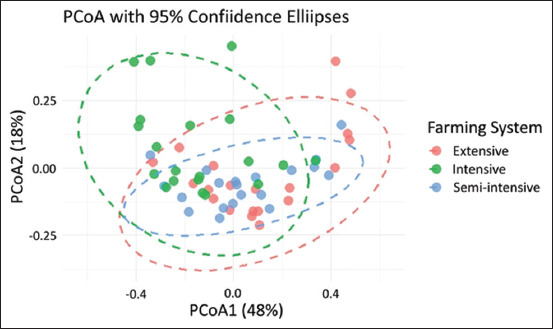
Principal component analysis (PCoA) plot based on Bray–Curtis dissimilarity showing health outcome distributions across farming systems with 95% confidence ellipses. The PCoA plot shows tighter clustering of semi-intensive farms, indicating greater health outcome consistency, while intensive and extensive farms display broader dispersion.

Figure 5 illustrates the multivariate distribution of animal health outcomes across various farming systems, using PCoA based on Bray–Curtis dissimilarity. The first two axes accounted for 66% of the total variation (PCoA1 = 48%, PCoA2 = 18%), enabling effective visualization of variability in health patterns among extensive, semi-intensive, and intensive systems. The extensive system (red circles) exhibited the greatest internal dispersion, indicating substantial variability in health outcomes among farms within this group. This variability may reflect diverse environmental exposures, differences in pasture quality, or variability in farming practices, which are often less standardized in extensive systems [13, 35]. In contrast, the intensive system (green) showed tighter clustering, suggesting greater consistency in health outcomes, likely due to more uniform infrastructure, controlled feeding regimes, and well-regulated environmental conditions [36]. However, despite this overall consistency, a few intensive farms displayed divergent health outcomes. This within-group variability may be attributed to differences in stockperson behavior, biosecurity measures, or farm management practices, even when structural conditions are comparable [32].

#### Joint effects of welfare levels and farming systems

**Figure 6:**
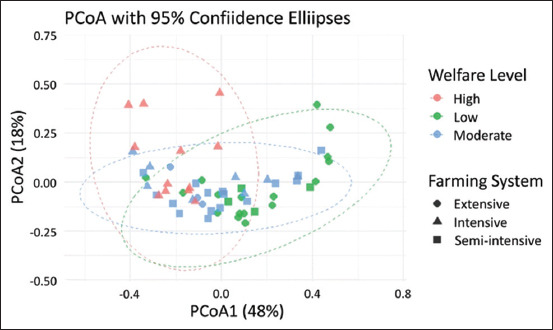
Principal component analysis plot based on Bray–Curtis dissimilarity showing health outcomes by welfare level (color) and farming system (shape) with 95% confidence ellipses, indicating partial separation of high-welfare intensive farms, tighter clustering of moderate-welfare semi-intensive farms, and greater variability among low-welfare farms across systems.

Figure 6 presents a two-dimensional PCoA plot that integrates both animal welfare levels (color) and farming systems (shape) to examine their joint effects on multivariate health outcomes. The first two axes (PCoA1 = 48%, PCoA2 = 18%) accounted for 66% of the total variation, allowing for a detailed visualization of how these factors interact. Farms with moderate welfare levels and semi-intensive production systems (blue squares) were generally closely clustered, indicating a high degree of similarity and consistency in health outcomes. This tight clustering suggests that mid-level welfare practices implemented within adaptable systems may produce steady and beneficial outcomes with relatively low resource input. Such a balance is particularly advantageous for smallholder farmers operating under resource constraints [37–40].

In contrast, the high-welfare group (red), particularly within intensive systems (triangles), exhibited a wider range of health outcomes. This suggests that high structural standards alone do not consistently translate into optimal health outcomes, likely due to variations in implementation, stockperson behavior, or unmeasured environmental factors, as noted by Mellor [41]. Although these farms may theoretically comply with welfare standards, actual day-to-day operations, such as monitoring, responsiveness, and the quality of human–animal interactions can differ significantly, resulting in varied outcomes [42, 43]. These findings highlight that infrastructure alone is insufficient; effective and attentive management is essential for achieving meaningful improvements in animal welfare.

The wide distribution of green low-welfare farms across all farming systems indicates substantial internal variability and instability in health outcomes, reinforcing the significant interaction effect shown in Table 6. This evidence suggests that neither welfare level nor farming system alone can fully explain health variation; instead, their interaction, along with consistent implementation, plays a more critical role [44].

**Table 6:** Two-way PERMANOVA results testing the effects of welfare level, farming system, and their interaction on multivariate health outcomes.

Effects	df	SS	R^2^	F	Pr(>F)
Main effects					
Welfare level	2	1.3386	0.1787	6.2017	<0.001
Farming system	2	1.0358	0.1382	4.5740	0.0003
Interaction effect					
Welfare level × Farming system	5	1.6117	0.2151	2.9610	0.0004
Residual	54	5.8786	0.7848		
Total	59	7.4904	1.0000		

PERMANOVA = Permutational multivariate analysis of variance, df = Degrees of freedom, SS = Sum of squares, R² = Coefficient of determination, F = F-statistic, Pr(>F) = Probability value associated with the F-statistic.

#### PERMANOVA results

A two-way PERMANOVA was conducted to statistically confirm these observed differences and quantify the proportion of variance explained by each main effect and their interaction on multivariate health outcomes in cattle (Table 6). The use of PERMANOVA, a non-parametric method that does not require multivariate normality, was particularly appropriate for this study, given the complex and often nonlinear nature of field data in livestock systems [45]. The results showed that welfare level had a statistically significant effect on health outcomes (F = 6.2017, R^2^ = 0.1787, p < 0.001), explaining approximately 17.87% of the total variation. The farming system also had a significant effect (F = 4.5740, R^2^ = 0.1382, p = 0.0003), accounting for 13.82% of the variation. These findings indicate that both welfare level and production system are significantly associated with livestock health outcomes, supporting previous research that links welfare conditions and management practices to animal health and productivity, particularly in smallholder and pasture-based systems [46, 47].

In addition, a statistically significant interaction was found between welfare level and farming system (F = 2.9610, R^2^ = 0.2151, p = 0.0004), indicating that animal health outcomes are influenced not only by either factor alone but also by their combined effect. This interaction accounted for the largest variation (21.51%), underscoring the importance of context-specific management that aligns welfare practices with the characteristics of each production system [38, 40]. These findings suggest that applying a uniform animal welfare approach across all farm types may be ineffective. Different farming systems manage animals, environments, and resources in distinct ways, resulting in varied health outcomes. Fraser *et al*. [32] emphasized that a complex interplay of factors, including housing, human–animal interactions, and daily management practices, shapes animal welfare. Recent studies further support the use of flexible, system-specific strategies as more effective for improving welfare across diverse farming contexts [13, 48].

### Policy implications and economic considerations

#### Role of welfare-oriented practices

Welfare-oriented practices become necessary to prevent health issues and improve overall herd well-being regardless of production systems. In addition to improving health outcomes, these improvements have economic implications for smallholder beef systems. Reduced incidence of diseases such as bloating, injuries, and parasitic infestations leads to lower veterinary costs, decreased mortality rates, and improved productivity. Various studies have demonstrated the importance of enhanced welfare, achieved through good housing, handling, and health management, which collectively led to improved production efficiency [49].

#### Economic trade-offs across systems

Beef cattle intensive systems typically have higher production costs than extensive systems. This is mainly attributed to the need for investment in infrastructure, housing, feed supplementation, labor, and veterinary care. In contrast, extensive systems are more dependent on natural resources, including pastures, and tend to require fewer additional inputs, reducing the direct costs of production. Nevertheless, the evident economic benefits of extensive systems are often countered by their increased susceptibility to diseases and less strict management practices, resulting in reduced animal health and productivity [13]. Semi-intensive systems provide a cost-effective balance between expenditure and health benefits. Pugliese *et al*. [50] reported that semi-intensive dairy farms provide better welfare, including improved ventilation, resting surfaces, and housing facilities, compared with intensive farms, despite both having similar health outcomes. Moreover, enhancing animal welfare can simultaneously improve both animal welfare and farm profitability. Fernandes *et al*. [49] stated that investments in animal welfare can result in business advantages, such as increased productivity, competitiveness, and risk mitigation, whereas Nkatekho [51] argued that the outcomes of better welfare can be higher animal health, decreased stress, increased productivity, and access to higher-value markets with increased profitability.

#### Policy alignment with national and regional standards

From a policy perspective, these findings align with the Animal Welfare Act (2014) and regulations by the Department of Livestock Development (DLD) [52, 53], which aim to advance welfare standards to enhance productivity, food safety, and animal health. In synergy with Good Agricultural Practices (GAP) and sustainable beef initiatives, welfare improvements at the farm level provide dual benefits for animal welfare and smallholder livelihoods. This study suggests that welfare-oriented practices provide evidence for policy interventions that reduce treatment costs, strengthen profitability, and support rural resilience. These findings are also consistent with international and regional standards. The World Organization for Animal Health (OIE) Terrestrial Animal Health Code identifies injury prevention, disease control, and adequate nutrition as central welfare principles [54]. Similarly, the Food and Agriculture Organization guidelines stress the role of stockpersonship, health management, and feeding practices as fundamental determinants of both animal welfare and productivity [55]. In a regional context, the Association of Southeast Asian Nations (ASEAN) Animal Welfare Action Plan (2017–2020) promotes consistency in the standards of livestock care to facilitate sustainable production and trade [56]. Our findings also resonate with field evidence from ASEAN smallholder systems, including Cambodia, Laos, Vietnam, and Indonesia, where similar challenges in feed quality, breed improvement, and animal health management have been reported [57–60]. The evidence suggests that the associations between welfare and health identified in our study are generalizable across tropical smallholder contexts, not only in Northern Thailand.

## CONCLUSION

This study demonstrated that animal welfare scores varied significantly across farming systems in Northern Thailand, with intensive farms achieving the highest welfare standards, semi-intensive farms occupying a moderate position, and extensive farms showing the lowest scores. Welfare domains such as Good Feeding, Good Housing, Good Behavior, and Stockpersonship differed markedly across systems, whereas Good Health did not vary significantly. Importantly, higher welfare levels were strongly associated with improved health outcomes, including lower mortality, reduced incidence of diarrhea, respiratory distress, bloating, parasitic infestation, and injuries, along with better BCSs. Multivariate analysis further revealed that both welfare level and farming system independently influenced health outcomes, but their interaction explained the greatest proportion of variance, underscoring the importance of system-specific welfare practices.

The findings highlight that animal welfare is not only an ethical concern but also a practical determinant of productivity and sustainability. Improving welfare through better feeding strategies, enhanced housing, preventive health care, and skilled stockpersonship can substantially reduce veterinary costs, lower disease burden, and improve herd performance. Semi-intensive systems, which balance resource inputs with consistent welfare outcomes, appear particularly promising for smallholder farmers, offering a feasible pathway toward sustainable production under resource constraints. These results also support the integration of welfare standards into national policies and regional frameworks, aligning with GAP, OIE, and ASEAN animal welfare initiatives.

A major strength of this study lies in its comprehensive design, which integrated a validated welfare assessment protocol adapted for Thai smallholder contexts with robust statistical analyses, including multivariate approaches such as PERMANOVA and PCoA. The study covered a full year of data collection, thereby accounting for seasonal variation and enhancing the reliability of findings. However, some limitations must be acknowledged. The cross-sectional design precludes causal inference, and unmeasured variables such as breed differences, herd size, and farmer-specific practices may also influence welfare–health associations. While the study achieved sufficient sample size to detect medium-to-large effects, the diversity of smallholder contexts suggests that further stratification is needed to capture finer-scale variation.

Future research should adopt longitudinal and interventional designs to establish causal pathways between welfare interventions and health outcomes. Studies stratified by herd size, breed, and farmer management style would provide greater insight into context-specific drivers of welfare and health. In addition, developing low-cost, farmer-friendly welfare assessment tools linked to decision-support systems could facilitate practical adoption at the farm level. Regional comparative studies across ASEAN countries would also help generalize findings and strengthen collaborative welfare improvement strategies.

In conclusion, this study provides strong evidence that animal welfare and farming systems interact to shape beef cattle health outcomes in Northern Thailand. Semi-intensive systems with moderate welfare levels emerged as a balanced and scalable model for smallholder farmers, demonstrating both health and economic benefits. The findings underscore that welfare-oriented practices are not merely ethical imperatives but strategic investments for improving livestock health, productivity, and farmer livelihoods. By embedding animal welfare into policy, practice, and farmer training programs, Thailand and similar regions can move toward more sustainable, resilient, and welfare-conscious beef production systems.

## DATA AVAILABILITY

Supplementary data are not publicly available but can be obtained from the corresponding author upon reasonable request.

## AUTHORS’ CONTRIBUTIONS

SS and NK: Conceptualized and designed the study, collected and analyzed the data, and drafted and edited the manuscript. PI, CS, ST, WI, and AW: Contributed to the study conceptualization and design and critically reviewed the manuscript. All authors have read and approved the final version of the manuscript.
